# Subcutaneous administration of a fusion protein composed of pertussis toxin and filamentous hemagglutinin from *Bordetella pertussis* induces mucosal and systemic immune responses

**DOI:** 10.22038/IJBMS.2018.29112.7026

**Published:** 2018-07

**Authors:** Ali Torkashvand, Fariborz Bahrami, Minoo Adib, Soheila Ajdary

**Affiliations:** 1 Department of Immunology, School of Medicine, Isfahan University of Medical Sciences, Isfahan, Iran; 2 Department of Immunology, Pasteur Institute of Iran, 69 Pasteur Ave., Tehran, Iran

**Keywords:** Bordetella, Filamentous hemagglutinin, Imiquimod, Pertussis, Pertussis toxin, Recombinant

## Abstract

**Objective(s):**

After decades of containment, pertussis disease, caused by *Bordetella pertussis* seems to be re-emerging and still remains a major cause of reported vaccine-preventable deaths worldwide. The current licensed whole-cell vaccines display reactogenicity while acellular vaccines are expensive and do not induce Th1-type immune responses that are required for optimum protection against the disease. Thus, there is an urgent need to develop new vaccines and the recombinant technology seems to be the method of choice for this purpose. The present study was an attempt to develop a new, simplified, cost-effective and well-defined vaccine against *Bordetella pertussis*, with capacity to induce a Th1 response.

**Materials and Methods:**

A fusion DNA fragment encoding the N-terminal region of pertussis toxin S1 subunit and filamentous hemagglutinin type 1 immunodominant domain was constructed and the corresponding fusion protein (F1S1) was produced in *Escherichia coli.* F1S1 in conjunction with imiquimod was administered by subcutaneous (SC) and intranasal (IN) routes to BALB/c mice.

**Results:**

This vaccine formulation could elicit high levels of IFN-γ, serum IgG (with higher IgG2a/IgG1 ratio) and lung IgA after the SC and, to a lesser extent, following the IN administration.

**Conclusion:**

Our results indicate that the above-mentioned important proteins of *B. pertussis* could be successfully produced in *E. coli *as a single fusion protein. Furthermore, this protein could induce proper systemic and mucosal immune responses after administration via SC or IN routes.

## Introduction

Whooping cough, also known as pertussis, is a highly contagious human respiratory disease. This ailment is a common cause of prolonged coughing and is accompanied with significant morbidity and mortality (1). The etiological agent of pertussis is a Gram-negative bacterium, named *Bordetella *(*B.*)* pertussis* that infects infants, young children, and even adolescents and adults. The bacterium produces several virulence-associated factors including adhesion molecules, namely filamentous hemagglutinin (FHA), fimbriae, and pertactin (PRN) as well as toxins such as pertussis toxin (PT), lipopolysaccharide (LPS), and the adenylate cyclase toxin, all of which can contribute to the pathogenesis and the symptoms observed during the infection (2, 3). Pertussis was controlled effectively following the introduction of the whole-cell pertussis (wP) vaccine in the 1940s. However, incidents of adverse reactions both at the local and systemic levels discouraged many countries from using wP, which led to outbursts of the disease. Such drawbacks steered the development and presentation of the acellular pertussis (aP) vaccine in the 1980s, consisting of a few defined purified proteins of *B. pertussis *(4). In addition to its reduced side-effects, the aP vaccine had also the advantage that it could be used for booster vaccination in adolescents and adults, which was not the case for the wP vaccine (4). Nevertheless, the high costs of production and supply chains of the aP vaccine have remained a major obstacle for the resource-limited countries. 

Several studies have pointed to the importance of induction of Th1-type immune responses, responsible for the cell-mediated immunity in protection against *B. pertussis *(5-7). 

 Similar to natural pertussis infection, the wP vaccine preferentially induces Th1 responses that favor cell-mediated immunity and is associated with protection against infection. In contrast, the aP vaccine induces Th2 responses that do not appear to be as effective as Th1 responses in clearance of *B. pertussis *from the respiratory tract (7-10). More recently, it has been reported that Th17 cells can also play an important role in protection (10, 11). 

PT, in its functional form, is exclusively found in *B. pertussis. *The Mw of the protein is 105 kDa, composed of five subunits, named as S1 to S5. The S1 subunit catalyzes the ADP-ribosylation of G proteins in its target mammalian cells. The detoxified form of PT is a protective component that is used in all aP vaccines (2, 12, 13). The chemical detoxification of PT, which is a part of the vaccine production procedure has been shown to destroy the protective epitopes that can consequently reduce the total immunogenicity and it may also be associated with the increased reversion rate of residual toxin activity (14). However, the genetically-detoxified PT, obtained by two amino acid substitutions (PT-9K/129G) within the S1 subunit, eliminates the enzymatic activity and the toxic properties of the wild-type toxin (15). The S1 subunit has been shown to be immunogenic and protective (13, 16) while the N-terminal-180 amino acid-segment of S1 is the most immunogenic and protective part (16).

FHA is another key virulence factor of *B. pertussis* and plays important roles in adhesion of the bacterium to the ciliated epithelium (2). Moreover, this protein is a potent immunogen and it has been shown to induce protective antibodies in animal models and humans (17, 18). FHA is incorporated in most aP vaccines to enhance the efficacy conferred by monocomponent PT toxoids (19, 20). FHA consists of two immunodominant domains, named type 1 and type 2, corresponding to carboxyl and amino termini of the protein, respectively. The type 1 immunodominant domain, corresponding to 456 amino acids from the carboxyl terminus has been shown to be the most immunogenic portion in both humans and mice (17, 18). Adjuvants are often necessary for the induction of a protective immune response against recombinant subunit antigens and protein toxins. Aluminum potassium sulfate, referred to in short as alum, is an adjuvant that is currently used in several human vaccines against infectious diseases including DTwP and DTaP. 

The purpose of the present study was to design a vaccine candidate against pertussis, composed of two immunogenic entities of *B. pertussis*, namely the immunogenic part of S1 subunit of PT fused to an immunodominant domain of FHA, formulated with IMQ as an adjuvant. Since alum is a poor inducer of Th1 responses, we chose imiquimod (IMQ), a TLR7 agonist that promotes both Th1-biased immune responses and antibody production and has been tested previously as a vaccine adjuvant for human dendritic cell activation (21).

## Materials and Methods

***Bacterial strains, growth conditions, and animals***

All cloning steps were performed in *Escherichia coli* BL21 (DE3), grown in Luria-Bertani (LB) broth containing ampicillin (100 μg/ml) at 37 ^°^C in a shaking incubator (220 rpm). *B. pertussis *Tohama I strain was grown at 35 ^°^C on Bordet-Gengou agar plates. Female BALB/c mice (4–6 weeks old) were purchased from the animal facility of Production Complex of Pasteur Institute of Iran in Karaj. All mice were kept under conventional conditions with water and food provided *ad libitum*. Experiments were performed in accordance with the guidelines of the Institutional Animal Care and Research Advisory Committee of Pasteur Institute of Iran.

***Gene synthesis and cloning of F1S1***

The nucleotide sequences encoding S1 subunit of PT and FHA were obtained from NCBI database (NCBI GenBank accession numbers AJ920066.1 and X52156.1, respectively). A sequence encoding 456 amino acids (residues 1656-2111) of FHA was designed to be linked to another sequence encoding the N-terminal 180 amino acid residues of S1 subunit via a Pro-Gln-Asp-Pro-Pro flexible linker (22). The required changes to create a biologically-inactive mutant S1 (PT-9K/129G) were implemented. To improve the codon-utilization, the encoding nucleotide sequence of the F1S1 fusion protein was optimized according to the codon frequency usage table for *E. coli*. The optimized sequence was constructed by a service provider (GeneCust, Luxemburg). The F1S1 gene (1926-bp) was amplified with f1s1F and f1s1R primers (Table 1). The resulting amplicon was double digested (*BamH*I and *Sac*I; Thermo Fisher Scientific) and was cloned in-frame into a *BamH*I- and *Sac*I-digested pET21a expression vector (Novagen, USA). 

***Expression and purification of the F1S1 fusion protein***

The verified recombinant construct (after restriction analyses and DNA sequencing reactions) was transformed into *E. coli BL21 *(DE3) for protein production by IPTG induction. The purification of F1S1 protein was performed by Ni-NTA resin (Qiagen, Germany) according to the manufacturer’s instruction under denaturing conditions with 8M urea. The protein was dialyzed overnight against several changes of PBS and endotoxins were removed using a Pierce High-Capacity Endotoxin Removal Resin spin column (Thermo Scientific, USA), according to the manufacturer’s recommendations. The protein concentration was determined by the Bradford method (23).

***SDS-PAGE and*** ***Western blot analysis of the*** ***F1S1 fusion protein***

The production and purity of F1S1 fusion protein were confirmed by SDS-PAGE and Western blotting analysis using anti-His (C-term)-HRP monoclonal antibody (Sigma, Germany) and a polyclonal antiserum raised against DTaP vaccine (Boostrix, GSK, Belgium) in mice. Horseradish peroxidase-labeled goat anti-Mouse IgG antibody (Sigma, Germany) was used as the secondary antibody. Finally, the blots were developed for visualization of the bands with DAB (3, 3′-Diaminobenzidine). To verify that the antibodies raised against recombinant protein can react with the epitopes on the natural proteins, antisera against DTaP (as a positive control) and F1S1 were used in a Western blot analysis to react with proteins of *B. pertussis* Tohama strain. 

***Immunization procedures***

Mice were randomized into 7 groups of 5 mice including: F1S1-IMQ-SC, F1S1- IMQ-IN, IMQ-SC, IMQ-IN, alum-SC, PBS-SC, and DTaP. For SC immunizations, 12 µg of F1S1 protein plus 20 μg of IMQ (ENZO, Germany); henceforth referred to as F1S1-IMQ, were injected (in 100 μl volume) in the mice tail base. IMQ was prepared with endotoxin-free water at 2 mg/ml in small aliquots and stored at -20 ^°^C until use. All mice were given 3 doses on days 0, 15, and 30. For IN immunizations, F1S1-IMQ (in 20 μl volume) was administered by pipetting into the mice nostrils for 3 consecutive days. This protocol was repeated twice with 2-week intervals. Control mice were immunized with IMQ via either SC or IN, alum or PBS via SC routes using the same schedule. The members of the seventh group of mice (i.e. the positive control group), were subcutaneously immunized with 1/4 of standard human dose (24) of a commercial DTaP vaccine (Boostrix, GSK, Belgium), containing 2 μg of the pertussis toxoid and 2 μg of FHA and 0.6 μg of PRN, using the same schedule as above.

**Table 1:** Primers used for PCR amplification and cloning of F1S1 Construct

Primer	(5ʹ–3ʹ)	Restriction enzyme
f1s1F (forward)	ACGTGGATCCTCACTTTACGCTGAACACGA	*BamH*I
f1s1R (reverse)	ACGTGAGCTCACTGAAGTGTATGGGTTTGG	*Sac*I

**Figure 1:**
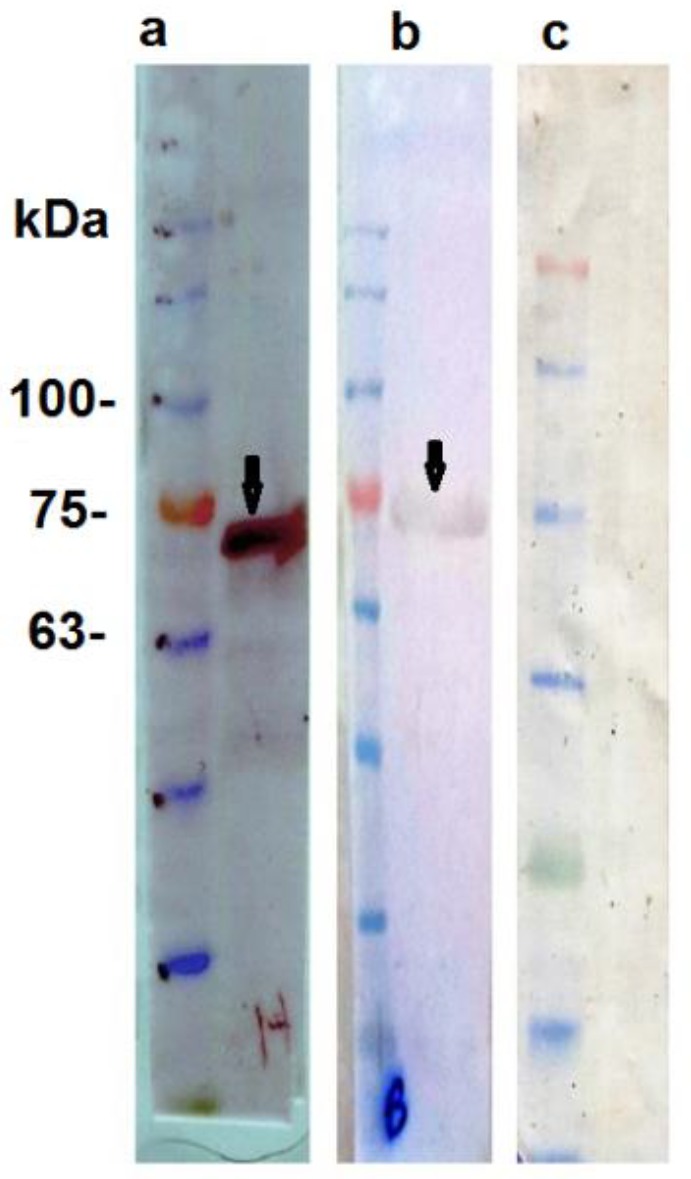
Western blot analyses of the F1S1 fusion protein. Detection of the fusion protein by (a) anti-His monoclonal antibody, (b) anti-DTaP antiserum, (c) normal mouse serum along with pre-stained Mw markers. Arrows indicate the F1S1 protein with Mw of ≈ 73 kDa

**Figure 2:**
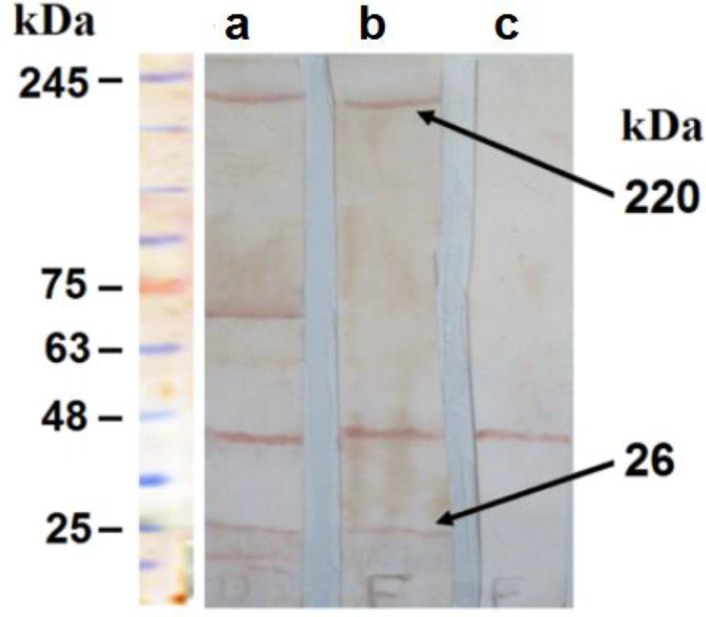
Verification of identical epitopes between F1S1 and natural FHA and S1. The whole cell lysate of B. pertussis strain Tohama I was examined by immunoblot analysis using (a) anti-DTaP antiserum, (b) anti-F1S1 antiserum, and (c) normal mouse serum along with a pre-stained Mw marker. Arrows indicate FHA (Mw of ≈ 220 kDa) and S1 (Mw of ≈26) proteins

**Figure 3:**
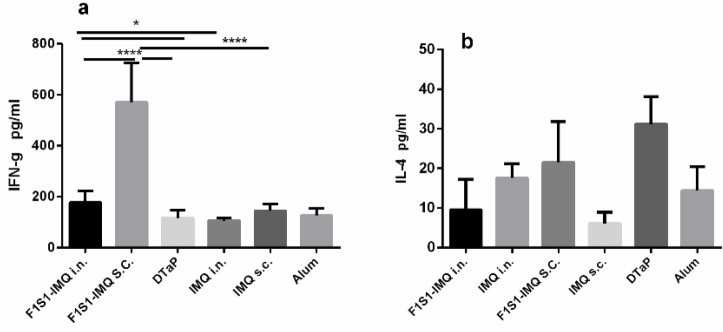
Cytokine responses, two weeks after the final immunization of BALB/c mice with recombinant F1S1 protein (via SC or IN routes) or commercial DTaP vaccine (via SC route). Mean + SD (pg/ml) of IFN-g (a) and IL-4 (b) concentrations in the supernatants of splenocytes of 5 mice per group after stimulation with F1S1 are shown (*P<0.05, **** *P*< 0.0001)

**Figure 4:**
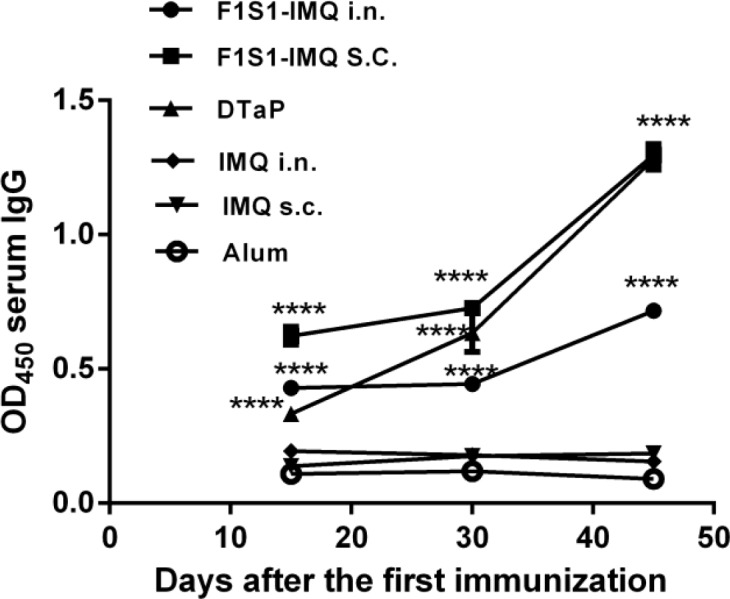
The kinetics of the F1S1-specific serum IgG antibody responses. Sera from 5 mice in each group were pooled and assayed in triplicates. The comparisons were made with adjuvant-treated control groups. (**** *P*<0.0001)

**Figure 5:**
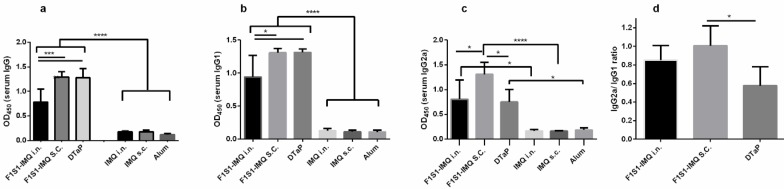
Anti-F1S1 specific antibodies in serum. Two weeks after the final immunization of BALB/c mice with recombinant F1S1 protein (via SC or IN routes) or commercial DTaP vaccine (via SC route), anti- F1S1 IgG (a), IgG1 (b), IgG2a (c) and IgG2a/IgG1 ratio (d) were assayed in sera by ELISA. Results depict A450 of sera diluted (1:100) and are expressed as mean + SD of 5 mice in each group (* *P*<0.05, *** *P*<0.001, *P*<0.0001)

**Figure 6:**
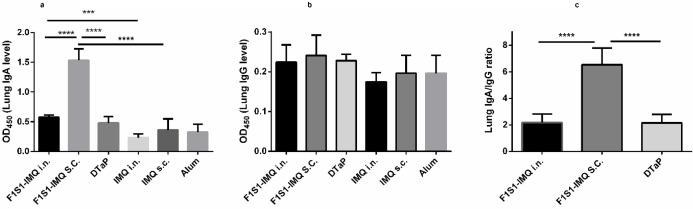
Specific anti-F1S1 antibody levels in lung homogenates. Two weeks after the final immunization of BALB/c mice with recombinant F1S1 protein (via SC or IN routes) or commercial DTaP vaccine (via SC route), anti- F1S1 IgA (a), IgG (b), and IgA to IgG ratio (c) were determined based on the measured secreted antibodies in the lung homogenates by ELISA. Results are expressed as mean + SD of 5 mice in each group (*** *P*<0.001, **** *P*<0.0001)

***Cytokine measurements***

The spleen from each mouse was removed aseptically, 2 weeks after the last booster. The splenocytes were isolated and adjusted to a concentration of 2 × 10^6^ per ml in an RPMI 1640 medium (Sigma, Germany), supplemented with 10% fetal calf serum (FCS; Sigma, Germany), 100 U/ml penicillin, 100 μg/ml streptomycin, and 2 mM L-Glutamine. The cells were incubated along with either 1 μg/ml of F1S1 or 2.5 μg/ml concanavalin A (Con A; Sigma, Germany), or only the medium as a negative control. The cultures were incubated for 72 hr at 37 ^°^C in a humidified 5% CO_2 _incubator and the supernatants were stored at -80 ^°^C for the cytokine measurements. IFN-γ and IL-4 were assessed by ELISA kits (eBioscience, USA), according to the manufacturer’s instructions.

***Evaluation of antigen-specific antibody levels***

Serum samples of the mice were obtained on days 0, 15, 30, and 45 after the first immunization. Two weeks after the last booster, mice were euthanized to prepare lung extracts for analysis of the immune responses. The lungs were harvested and briefly washed with PBS, ground gently into 0.5 ml cold PBS individually and were then passed through steel mesh filters. The resulting suspension was clarified by centrifugation at 5000 **× ***g*, 4 ^°^C for 15 min and the supernatants were stored at -20 ^°^C until use. The serum of each individual mouse was assayed for F1S1-specific IgG, IgG1, and IgG2a antibodies by ELISA. Similarly, the lung extracts of each individual mouse were assayed for IgG and IgA anti-F1S1 antibodies. In brief, ELISA plates (Greiner, Germany) were coated with 280 ng purified F1S1 and blocked by 1% BSA in PBS (BSA-PBS). The diluted sera (1:100) or the lung extracts (1:50) were added to the wells. Goat anti-mouse IgG, rabbit anti-mouse IgG1, rabbit anti-mouse IgG2a, or goat anti-mouse IgA antibodies (Sigma, Germany), all conjugated to horseradish peroxidase, were used as the secondary antibodies. The plates were developed with 3, 3′, 5, 5′-Tetramethylbenzidine (TMB; Sigma, Germany) and read at 450 nm. 

***Statistical analysis***

One-way analysis of variance (Multiple comparison Tukey’s *post hoc* test) was performed using GraphPad Prism 6.0 for Windows (GraphPad Software Inc, San Diego, CA, USA). *A P-*value < 0.05 was considered to be significant. 

## Results

***Characterization of recombinant protein***

Following the induction with 1.5 mM IPTG for 4 hr, the recombinant protein with a theoretical Mw of ≈ 73 kDa was highly produced. The total yield of the fusion protein from 1 liter of the induced culture was approximately 10 mg. The production of the F1S1 fusion protein was further confirmed by immunoblotting using sera from mice that had been immunized with the DTaP vaccine and anti-His tag monoclonal antibody (Figure 1). Furthermore, conservation of epitopes on the recombinant F1S1 protein was verified by binding of anti-F1S1 antiserum to *B. pertussis* strain Tohama I proteins in a Western blotting assay. Anti-DTaP antiserum was used as a positive control. As depicted in Figure 2, anti-F1S1 antiserum reacted with two proteins with Mw of ≈ 220 and 26 kDa, corresponding to Mw of *B. pertussis* natural FHA and S1 proteins, respectively. These results indicated that the F1S1 recombinant protein could induce antibodies, capable of interacting with both S1 and FHA proteins in their natural forms.

***Cytokine responses of splenocytes from the immunized mice***

Two weeks after the last booster, the concentration of secreted IL-4 and IFN-γ upon *in vitro* stimulation of the spleen cells with F1S1 protein was determined by ELISA. The mice that received PBS did not produce any cytokine; while a large comparable amount of IL-4 and IFN-γ was produced when spleen cells from all groups were stimulated with ConA (data not shown), indicating that the assay conditions for cytokine measurement were satisfactory. The F1S1-immunized groups produced significant amounts of IFN-γ, compared to their respective adjuvants groups (*P*<0.05, Figure 3a). The mice that were immunized subcutaneously with F1S1-IMQ and those immunized intranasally with F1S1-IMQ showed significantly higher levels of F1S1-specific IFN-γ secretion, compared to the DTaP group. The difference observed between the two F1S1-IMQ-immunized groups (SC vs. IN) was also significant (*P*< 0.0001). In response to stimulation with F1S1 protein, the splenocytes from DTaP-immunized group did not produce a significant amount of IFN-γ, compared to splenocytes from the mice that had received alum alone. Regarding IL-4 secretion, there was no statistically significant increase in IL-4 secretion in any of the studied groups, even though the mice that were immunized with commercial DTaP vaccine showed a relatively-higher IL-4 production (Figure 3b).

***Antigen-specific antibody responses***

Sera were collected on days 0, 15, 30, and 45 after the first immunization and tested for the induction of specific antibodies against the F1S1 protein. No anti-F1S1-specific antibody was detected for the PBS group. The IgG antibody responses in F1S1-IMQ-inoculated mice were measured and compared with those in either DTaP- or adjuvant-treated mice. Significant amounts of anti-F1S1 IgG antibodies were detected in the sera of all immunized groups on day 15, compared to their respective controls (*P*<0.0001). After a booster injection, IgG levels were significantly increased in mice immunized by either F1S1 via SC route or the DTaP group, compared to the results of day 15 (*P*<0.0001, Figure 4). 

As shown in Figure 5, on day 45 (two weeks after the final immunization), significantly higher amounts of specific IgG, IgG1 (*P*<0.0001), and IgG2a antibodies (*P *<0.0001 for the SC group and *P*<0.05 for IN and DTaP groups) were detected in the immunized mice, compared to their respective control groups. Figure 5a shows that the level of anti-F1S1 IgG in sera from SC immunized mice was comparable to that of the DTaP group. This level was significantly higher than the values obtained from the group that was immunized intranasally (*P*< 0.001). Moreover, anti-F1S1 IgG1 level in mice that were immunized intranasally with F1S1 was significantly lower than the level in mice immunized with F1S1 by the SC route and the DTaP group (*P*<0.05, Figure 5b). This level was comparable between the two latter groups. However, mice that were immunized subcutaneously with F1S1, had significantly higher amounts of IgG2a, compared to IN and DTaP groups (*P*<0.05, Figure 5c). The IgG2a/ IgG1 ratio was the highest in the group that was immunized with F1S1 by the SC route, followed by IN and DTaP groups; however, the difference was significant only between SC and DTaP groups (*P*<0.05, Figure 5d). 

Mucosal anti-F1S1 IgA levels were also determined in the lung extracts from the different groups, two weeks after the last immunization. Significant levels of anti-F1S1 IgA were detected in F1S1-immunized groups either by SC (*P*<0.0001) or IN (*P*<0.001) routes in comparison to their respective adjuvant controls. Interestingly, the highest anti-F1S1 IgA responses were observed in the lung homogenates of mice that were immunized subcutaneously with F1S1, which were significantly higher than those of IN and DTaP groups (*P*<0.0001, Figure 6a). No significant differences in induction of mucosal anti-F1S1 IgA responses could be detected in the lung homogenates of mice immunized with DTaP vaccine, IMQ, and alum. The analysis of the lung homogenates collected from the same mice revealed that there was no obvious F1S1-specific IgG in the lung homogenates of F1S1- and DTaP-immunized groups (Figure 6b). The IgA/IgG ratio was significantly higher in the group that was immunized with F1S1 by the SC route, compared to IN and DTaP groups (*P*<0.0001, Figure 6c).

## Discussion

It is well-documented in several studies that immunization with wP vaccines and infection with *B. pertussis *induce primarily mixed Th1/Th17 responses while immunization with aP vaccines induces Th2/Th17 responses (5-7). Nonetheless, it has been shown that the optimum protection against *B. pertussis* requires induction of Th1 cells (10, 11). In the present study, for development of a new, simplified, cost-effective and well-defined vaccine against *B. pertussis*, capable of Th1 response induction, we designed and constructed a fusion protein (F1S1), composed of the S1 subunit of PT plus FHA of *B. pertussis*. The N-terminally truncated S1 subunit was used since this region has the highest capability to induce protective immunity whereas the hydrophobic C-terminal part of S1 has a destabilizing effect when expressed in the cytoplasm of the heterologous hosts (15, 16, 22). Likewise, most of the reactive epitopes and cell-binding sites of FHA are located in its type I immunodominant domain of FHA, which elicits protective responses against *B. pertussis *infection (18).

F1S1 was successfully produced and was identified by Western blotting assays. Our data indicated that natural PT and FHA were recognized by anti-F1S1 IgG and antiserum against natural PT and FHA reacted with recombinant F1S1. These findings suggested the conservation of at least some of the epitopes of the natural proteins in F1S1. Moreover, the results indicated that F1S1 recombinant protein induces antibodies against both S1 and FHA proteins. These results were in line with a previous study on an almost similar fusion protein, named FsmS1. However, in that study, the sequence was not codon-optimized and its Fs portion consisted of the last 138 amino acid residues at the C-terminal of FHA type I domain, which was much shorter while FsmS1 had only been used to raise antibody against FsmS1 in rabbits without any further analysis (22).

The vaccine adjuvant activities of IMQ have been reported in several studies (21, 25). In order to promote an appropriate immune response and to enhance the *potency of the vaccine candidate,* the F1S1 fusion protein was formulated with IMQ adjuvant. Furthermore, since *B. pertussis *enters and colonizes the body through the respiratory tract, an ideal vaccine for our purpose should not only be capable of stimulating of specific IgG, but it also should be able to provoke IgA secretory antibodies. Therefore, different groups of BALB/c mice were immunized with F1S1-IMQ via SC, as well as IN routes. According to our results, the highest levels of F1S1-specific IFN-γ secretion were detected for splenocytes, isolated from BALB/c mice that were immunized subcutaneously with F1S1-IMQ, followed by those immunized intranasally, albeit at a lower level compared to the adjuvant controls. Consistent with other studies, no significant level of IFN-γ was detected in splenocytes of mice that were immunized subcutaneously with a commercial DTaP vaccine, (26). Although this group showed elevated levels of IL-4 production, there was no statistically significant increase in IL-4 secretion in any of the groups. Altogether, high levels of IFN-γ and non-significant levels of IL-4 in the mice that were immunized with F1S1-IMQ via SC or IN routes suggested the induction of a Th1-dominant response while the cytokine pattern in DTaP-vaccinated mice was indicative of a dominant Th2 type response. 

Our results also showed the highest levels of anti-F1S1 IgG in mice immunized subcutaneously with F1S1-IMQ, followed by those immunized with DTaP and the IN groups. The titers of IgG2a and IgG1 are known indicators of Th1 and Th2 responses in mice, respectively. Further analysis of the sera for both specific IgG1 and IgG2a isotype antibodies against F1S1 revealed that mice immunized with the commercial DTaP vaccine and with F1S1-IMQ had the lowest and the highest ratios of IgG2a/IgG1, respectively. These findings further confirmed the Th2 type dominance in the DTaP-vaccinated group and the Th1 type dominance of the immune responses in the F1S1-IMQ group. These observations were consistent with the known properties of the adjuvants, in that IMQ has a tendency to the Th1 responses whereas alum favors the Th2 responses (27-29).

Previous studies have indicated that local secretory antibodies such as IgA play roles in anti-*B. pertussis* immune responses (30, 31). Therefore, we evaluated whether immunization with a recombinant protein was able to induce mucosal antibody responses. Lung homogenates of the animals were tested for detection of IgG and IgA. Surprisingly, the highest anti-F1S1 IgA responses were observed in the lung homogenates of mice that were immunized subcutaneously with purified F1S1-IMQ. To a lesser extent, mice immunized intranasally with F1S1-IMQ, revealed significantly higher anti-F1S1 specific IgA levels, compared to the mice that had received IMQ alone. Moreover, no significant induction of mucosal anti-F1S1 IgA responses could be detected in the lung homogenates of mice immunized with the DTaP vaccine, indicating the inability of this formulation with respect to mucosal immune stimulation. This was in keeping with the findings of Knight *et. al. *who have shown that parenteral immunization with recombinant FHA adsorbed on alum did not produce detectable specific IgA in saliva (18).

Despite the general belief that parenterally-delivered vaccines fail to induce immune responses in the mucosal tissues, evidence has shown the likelihood of induction of mucosal immune responses after systemic vaccinations. Clements and Freytag have reviewed and discussed this subject in a recent article and have suggested a role for some of the TLR agonists in the induction of the mucosal immunity, following parenteral administrations (32). Our data in which high IgA levels in lung homogenates of mice that were immunized subcutaneously with F1S1-IMQ were demonstrated, confirmed this suggestion.

## Conclusion

Our results for the first time demonstrated that a double-component pertussis vaccine could be successfully produced in *E. coli* as a single fusion protein with a proven immunogenicity via both systemic and mucosal administrations. Immunization with F1S1 fusion protein induced specific serum IgG and lung IgA antibodies as well as specific T cell responses of the Th1 subpopulation that could be considered as the proper responses against pertussis. Furthermore, the high yield of production of recombinant F1S1 protein in *E. coli* was another advantage of this putative vaccine, which potentially makes it a new generation of cost-effective aP vaccines against pertussis, especially for the developing countries. Altogether, the present study is a promising proof-of-principle that needs further elaboration to evaluate its full potentials. 
